# Heavy metals contamination in sediments of Bharalu river, Guwahati, Assam, India: A tributary of river Brahmaputra

**DOI:** 10.1371/journal.pone.0283665

**Published:** 2023-04-05

**Authors:** Rajashree Bhuyan, Pinki Brahma, Mayuri Chabukdhara, Neha Tyagi, Sanjay Kumar Gupta, Tabarak Malik

**Affiliations:** 1 Department of Environmental Biology and Wildlife Sciences, Cotton University, Guwahati, Assam, India; 2 Department of Civil Engineering, Indian Institute of Technology, Hauz Khas, New Delhi, India; 3 Department of Biomedical Sciences, Institute of Health, Jimma University, Jimma, Ethiopia; Alagappa University, VIET NAM

## Abstract

This study aimed to assess heavy metals in the surface sediments of the Bharalu river, India. Metal concentrations ranged from 6.65−54.6 mg/kg for Ni, 25.2−250.0 mg/kg for Zn, 83.3−139.1 mg/kg for Pb, and 11940.0−31250.0 mg/kg for Fe. The level of metal contamination was assessed using sediment quality guidelines, geo-accumulation index (I_*geo*_), enrichment factor (EF), pollution Load Index (PLI),Nemerow’s pollution index (PIN), and potential ecological risk index. Pb exceeded the sediment quality guidelines at all sites indicating a potential threat to the river ecosystem. (I_*geo*_) and EF also showed moderate to severe enrichment for Pb. Potential ecological risk (RI) showed low risk in the sediments, and Pb is the major contributor to ecological risk. Overall, pollution indices revealed comparably higher contamination of the sediments in the downstream sites than in the upstream site. PCA and correlation matrix analysis indicated both anthropogenic and natural origins for metals. Among anthropogenic sources, urban discharges and waste dumping could be mainly attributed to metal contamination in the river sediments. These findings may aid in developing future river management methods explicitly aimed at tackling heavy metal pollution to prevent further damage to the river ecosystem.

## 1. Introduction

More than half of the world’s population lives in cities, and understanding the processes that affect urban systems is of global concern [1,2]. Urban rivers are vulnerable to exogenous pollution due to the relatively restricted surroundings of narrow watersheds, complex flow zones, and delayed water rejuvenation [3]. Sediments have long been perceived as a critical indicator of water contamination [4,5] as sediment has a high contaminant retention capacity and also releases accumulated contaminants back into the river (water) system [6].

Among several contaminants, heavy metal pollution of the surface water bodies has drawn special consideration due to their non-biodegradable nature, bioaccumulation capacity, and food chain contamination [7]. As disposal sites for various industrial and urban treated and untreated effluents, river sediments are most sensitive to metal contamination [8]. Metals contamination in surface sediments along urban rivers has recently been a major concern. Urban and industrial activities have been linked to heavy metals in urban river sediments and associated ecological risks [9–11]. The migration of metals from sediments to overlying water and other environments leads to severe environmental and human health risks [12].

Surface soil’s metals can come from natural and multiple anthropogenic sources [13]. Point sources of pollutants in urban river sediments must be evaluated and identified regularly to reveal the impact of city expansion on the river ecosystem [13]. Heavy metal contamination in aquatic sediments has a longer residence time, which may aid in more effective monitoring of pollution [14].

The Bharalu river is one of the tributaries of the Brahmaputra river that passes through densely populated areas of Guwahati city, Assam, India, until its confluence with the Brahmaputra at Bharalumukh. Very few studies have been reported earlier on Bharalu river water quality [15,16] and sediments [17] in Guwahati city. Guwahati is a city in northeast India that is rapidly expanding. Guwahati and its environs have seen tremendous development and urbanization in the past few decades, severely affecting the Bharalu ecosystem. Therefore, the current research was conducted with the following objectives: (1) to investigate the level of heavy metal contamination in the sediments of Bharalu river (2) to assess the extent of contamination in terms of enrichment factor and geo-accumulation index, pollution load index, and Nemerow’s Pollution Index (3) to assess the possible sources of metals in river sediments.

## 2. Materials and methods

### 2.1. Study area

The Bharalu iver emerges in Meghalaya, India, amid the foothills of the Khasi Hills. It flows through Guwahati’s industrial and commercial center before its confluence with the river Brahmaputra at Bharalumukh. The river Brahmaputra in the city is utilized as a water resource for several purposes, including serving as a principal source of consumptive water. Guwahati city is located at 24.1445°N, 91.7362°E. Bharalu has a drainage area of roughly 120 km^2^, draining approximately 10.94 km^2^ of Guwahati city [18]. The city rests upon Precambrian rock covered by young alluvium [19].

The total population in 2011 was 957,352 and had the highest population density in northeast India, with 4400 persons per square km [20]. Therefore, as one of the Brahmaputra’s tributaries, preserving the water quality of the Bharalu river is crucial.

### 2.2. Sampling, monitored parameters, and analytical methods

**Fig 1:**
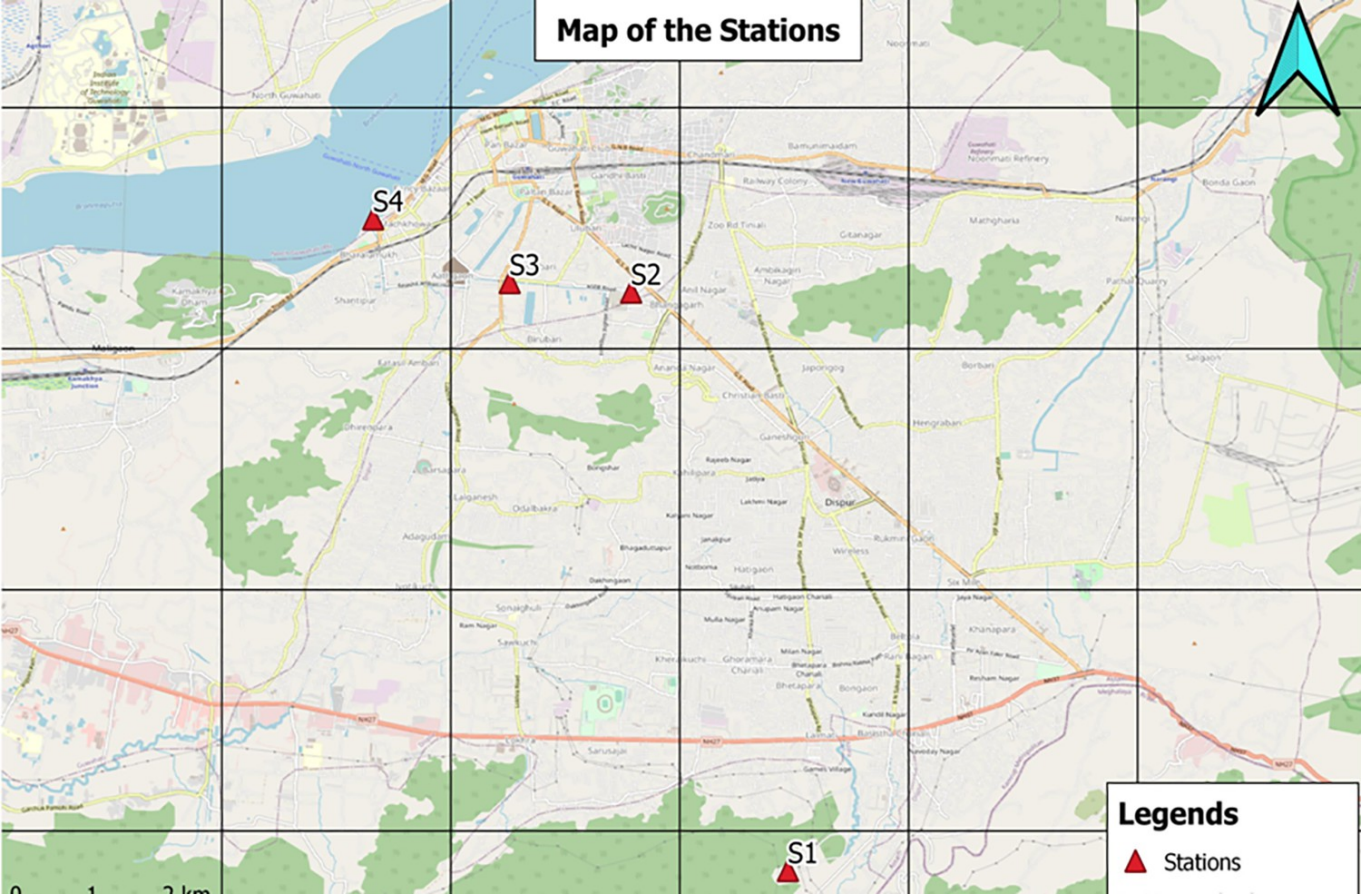
Map showing sampling locations.

In the present study, four sites, namely Basistha (S-1), Bhangagarh(S-2), Sharabhatti (S-3), and Bharalumukh (S-4), were selected (Fig 1) for sampling of river sediments. Sampling was done in April 2019. At each site, three surface sediment samples (0–20 cm) were collected with a stainless steel auger, immediately placed in polypropylene zipped pouches, and brought to the laboratory. A GPS recorded the location (longitudes and latitudes) of each site. No specific permission was required for sampling from the river Bharalu. The study area is not privately-owned or protected in any way, and the field studies did not involve endangered or protected species.

Air-dried sediment samples were sieved through a mesh (< 2 mm). The pH of the collected soil samples (1:5 w/v) was measured with a pH meter, and the electrical conductivity was determined using a conductivity meter. Soil organic carbon was estimated following Walkley and Black method [21]. The US E.P.A technique 3050B was used for digesting sediments with HNO_3_∕H_2_O_2_∕HCl combination for metal analysis [22]. The levels of toxic metals in sediment samples were determined with atomic absorption spectrometry (model 4129, E.C.I).

### 2.3. Quality assurance and quality control

Chemicals of analytic grade (A.R.) were utilized during the study without any additional purification. All reagents and calibration standards were made with ultra-pure water. All of the tests were performed three times. Procedure blank samples and reagent blanks were applied across the entire process. A standard reference material (SRM 2711) was used to ensure the accuracy of the determinations, and the recoveries were 90%-108%.

### 2.4. Data analysis

A statistical tool, SPSS^®^ (Windows Version 17.0), was used to examine the data. Descriptive statistics include mean, range and standard deviation. Depending on the data type, various datasets have been subjected to correlation analysis. Principal component analysis (PCA) was applied for source analysis of heavy metals in sediments. The details of PCA used for data analysis are mentioned elsewhere [8]. Before multivariate analysis, Shapiro-Wilk’s normality test (*p*> 0.01) was used to determine the normality of the data. Logarithmic transformation was used to normalize the original data.

### 2.5 Geo-accumulation Index (I_geo_) and Enrichment factor (E.F.)

The Geo-accumulation index (*I*_*geo*_) facilitates comprehensive contamination assessment by comparing contemporary and pre-industrial values. In this study, the *I*_*geo*_ for sediment samples was determined using the following equation [23]:

$$I_{\mathrm{geo}}=\log_{2}(C_{n}/1.5B_{n})$$ (1)

where C_n_ = measured concentration, mg/kg and B_n_ = geochemical background value, mg/kg. In Eq 1, average values were used, and 1.5 is the factor used for lithologic variations of trace metals. The values of the geo-accumulation index categorization described by Forstner et al. [24] are compared to the geo-accumulation index results obtained for the sediment (S1 Table).

The Enrichment Factor (E.F) is a geochemical index method used to evaluate the influence of anthropogenic practices on heavy metal levels in sediments. Generally, an anthropogenic origin of the component of concern is indicated by an enrichment factor larger than one [25]. The E.F is described as follows [26],

$$EF={\left(Me/Fe\right)}_{\mathrm{Sample}}/{\left(Me/Fe\right)}_{\mathrm{Background}}$$ (2)

where (Me/Fe)_Sample_ is the metal to Fe ratio in the samples of interest; (Me/Fe)_Background_ is the geochemical background value of the metal to Fe ratio. In this study, we used geochemical average shale values [27] as background values, which are 68.0 for Ni, 95.0 for Zn, 20.0 for Pb, and 46700.0 for Fe. As Birth [28] recommended, the E.F values were discussed in the context of heavy metal contamination (S1 Table).

### 2.6 Pollution level assessment using pollution indices

The Pollution Load Index (PLI) and Nemerow’s pollution index (PIN) are two extensively used methods for assessing metal pollution load in sediments. The PLI scale is used to evaluate the extent of metal pollution. The following equation was used to determine PLI [29]:

$$PLI={\left(CF_{1}\times CF_{2}\times CF_{3}\times \ldots \ldots \ldots CF_{n}\right)}^{1/n}$$ (3)

Where PLI is the Pollution Load Index; C.F is the contamination factor and is calculated by following the Håkanson method [30]:

$$CF=\frac{C_{s}}{C_{b}}$$ (4)

Where C_s_ represents the observed level of heavy metal in sediment and C_b_ represents the metal’s baseline level in sediment.

A number of 0 on the PLI, implies that no contaminants are present; a value of 1 shows that baseline contamination levels are present, and values above 1 indicate that the sampling locations are deteriorating [29]. For the integrative assessment of sediment metals, Nemerow’s pollution index was derived as per the following equation [31]:

$$PIN=\sqrt{\frac{{\left({CF}_{iavg}\right)}_{}^{2}+{\left({CF}_{iavg}\right)}_{}^{2}}{2}}$$ (5)

Where PIN is Nemerow’s pollution index, CF_*iave*_ is the average CF value of each heavy metal, and CF_*imax*_ is the maximum *CF*. value of each heavy metal. The PIN was classified as [32]: clean (PIN ≤ 0.7); warning limit of pollution (0.7 ≤ PIN ≤ 1); slight pollution (1 ≤ PIN ≤ 2); moderate pollution (2≤ PIN ≤ 3) and heavy pollution (3≤ PIN).

### 2.7 Ecological risk assessment

The following formulae were used to determine RI [30,33]:

$$c_{f}^{i}=c_{s}^{i}/c_{n}^{i}$$ (6)

$$E_{r}^{i}=T_{r}^{i}*c_{f}^{i}$$ (7)

$$RI=\sum E_{r}^{i}=\sum T_{r}^{i}\times c_{f}^{i}$$ (8)

where $c_{f}^{i}$ is the contamination coefficient, $c_{s}^{i}$ is the determined concentration of heavy metal i, and $c_{n}^{i}$ is the background value of heavy metal ‘i’ in sediments. $T_{r}^{i}$ is the toxicity coefficient; its values for Pb, Zn and Ni are 5, 1 and 5, respectively [34]. The RI signifies the potential ecological risk induced by cumulative contamination, which is the computation of all risk coefficients for toxic metals, and $E_{r}^{i}$ reflects the individual ecological risk of metal *i*. The values of $E_{r}^{i}$ values can be categorized into five different types (S2 Table), whereas the RI values can be separated into four classes [30].

## 3. Results and discussion

### 3.1 Physiochemical characteristics and heavy metals in river sediment

**Fig 2:**
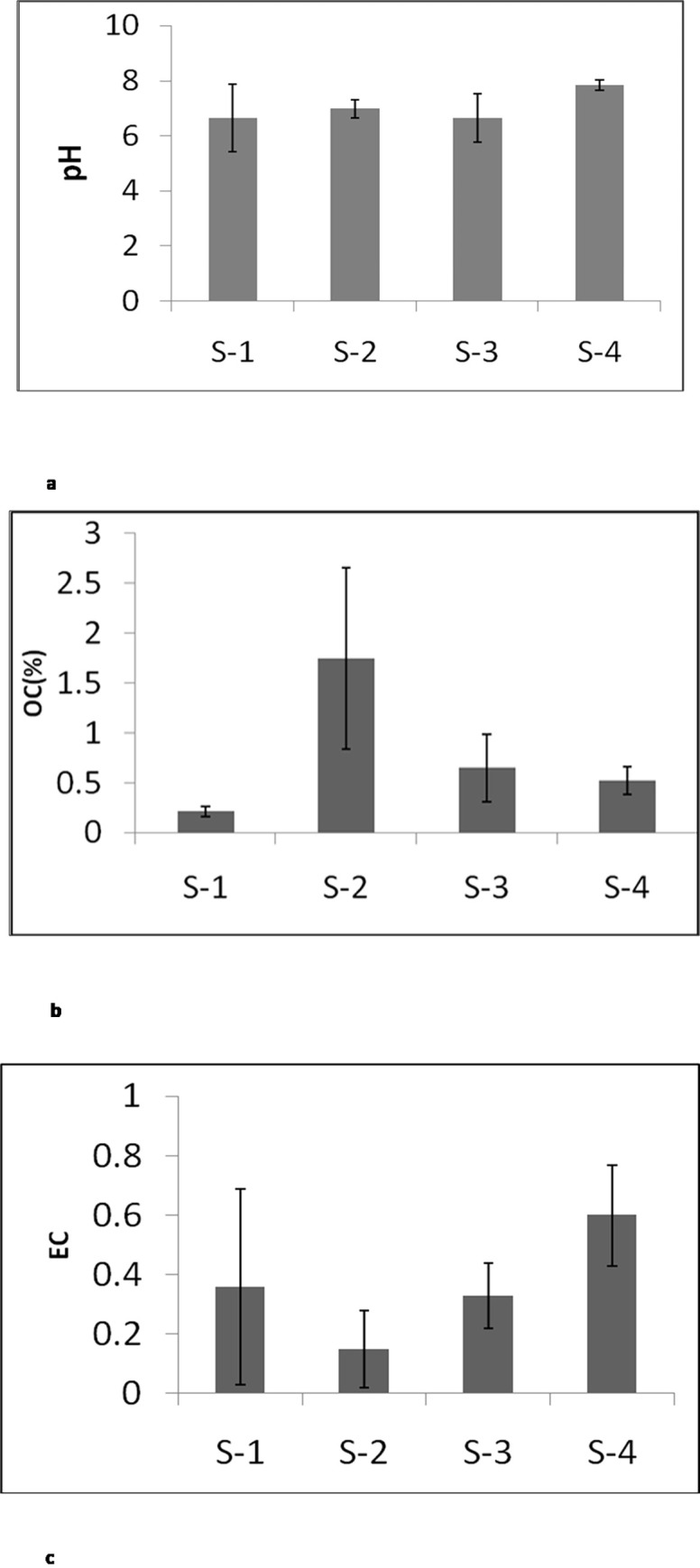
pH (a), EC (b) and OC (c) level in sediments from different sites.

The pH of all sediments samples ranged from 5.9 to 8.05, indicating that they were slightly acidic to alkaline (Fig 2A). The organic matter content in sediment ranged from 0.2% to 1.96%, and the electrical conductivity ranged from 0.06 to 0.8 mS/cm (Fig 2B and 2C). Table 1 shows a statistical overview of toxic metals in sediment at various sites along the Bharalu river. Considering all sites, metal concentration in river sediments in Bharalu ranged (mg/kg): Ni (6.65−54.6), Zn (25.2−250.0), Pb (83.3−139.1), and Fe (11940.0−31250.0).

**Table 1:** Statistical summary of heavy metals (mg/kg) in river Bharalu sediments.

Site		Ni	Zn	Pb	Fe	
S-1	Mean	8.08	31.87	91.93	12890.67	
	SD	1.87	5.82	5.67	955.03	
	Min	6.65	25.20	86.25	11940.00	
	Max	10.20	35.90	97.60	13850.00	
S-2	Mean	26.77	242.83	124.27	19589.67	
	SD	5.88	7.01	20.87	1328.18	
	Min	20.10	236.00	100.40	18142.00	
	Max	31.20	250.00	139.10	20752.00	
S-3	Mean	43.23	75.40	116.70	15265.00	
	SD	10.57	10.60	7.01	852.59	
	Min	33.70	63.20	111.40	14420.00	
	Max	54.60	82.40	124.65	16125.00	
S-4	Mean	39.37	85.87	92.83	30447.33	
	SD	15.12	9.90	8.72	754.75	
	Min	22.30	75.80	83.30	29752.00	
	Max	51.10	95.60	100.40	31250.00	
TEL (threshold effect levels)		35	123	18	-	[35]
PEL (probable effect levels)		91.3	315	36	-	[35]

Overall mean concentrations of metals were (mg/kg): 29.36 for Ni, 108.9 for Zn, 106.43 for Pb, and 19548.2 for Fe. The mean concentrations of HMs in river sediment were ranked as Fe > Zn > Pb> Ni. Heavy metals levels in the river Bharalu are compared to those in other Indian rivers (S3 Table).

As can be seen, the mean Pb level (106.43 mg/kg) was higher than in many other rivers in India. A higher mean Pb level (169.8 mg/kg) was reported in Deepor Beel, a Ramsar site and of the largest freshwater lakes of the Brahmaputra Valley, located in Guwahati city, Assam, India [36]. The maximum Pb content in the current study is lower than that reported in Kinshasa City, Congo urban rivers and other urban rivers across the world [7], Langat River, Malaysia [5], river Hindon, India [8], and urban river sediments in China [10,13,37] but was higher than those reported in river Couvary, India [38], river Ganga, India [39], urban river sediments in China [1,33,40].

Similarly, the maximum concentration of Zn reported in the river Bharalu was lower than those reported in urban rivers of Kinshasa City, Congo [7], river Couvary, India [38], Turag River, Bangladesh [41], urban river sediments, China [1,10,33,40,42] but was higher than those reported in Langat River, Malaysia [5] and river Ganga, India [39]. The maximum Ni level was lower than those reported in river Hindon, India [8], and urban river sediments in China [7,37,42]. But the maximum Ni level in the present study was higher than those reported in Couvary, India [38], river Ganga, India [39], and urban river sediments in China [40]. The highest Fe level in the river Bharalu was lower than those reported in the Turag River, Bangladesh [41] and river Ganga, India [39] but was higher than those reported in the river Hindon [8].

When compared to eco-toxic threshold values for metals in the sediment [35] in terms of Threshold Effect Level (TEL) and Probable Effect Level (PEL), the mean level of Ni and Zn was found to be within the limit (Table 1). However, the Pb value was far higher than the threshold effect level (TEL) of 18 mg/kg and the probable effect level (PEL) of 36 mg/kg. Further, the mean value of Pb exceeded the TEL and PEL at all sites. An average level of Ni outstripped the TEL of 35 mg/kg at S-3 and S-4, while the mean Zn exceeded the TEL of 123 mg/kg at S-2. The results indicate that Pb and Ni level in the sediment is a major concern for the Bharalu river as a freshwater ecosystem. S-2 and S-3 pass through the city’s dense part that receives urban wastes and discharges. S-4 is the site that opens at the river Brahmaputra.

### 3.2 Pollution levels of metals in sediment

Table 2 shows the *I*_geo_ and EF of metals in the sediment of the Bharalu river. The mean *I*_geo_ values of toxic metals in the sediment samples were ranked as: Pb (1.81) > Zn (-0.77)> Fe (-1.92)> Ni (-2.09). Table 2 shows Ni, Zn and Fe remained in class 0 (practically uncontaminated). Pb showed moderate contamination at S-1, S-3, and S-4 (class 2) and moderately strong contamination at S-2 (class 3).

**Table 2:** Geo-accumulation index (I_*geo*_) and Enrichment factor (EF) of different metals in river sediments.

I_*geo*_					EF		
	Ni	Zn	Pb	Fe	Ni	Zn	Pb
S-1	-3.68	-2.18	**1.57**	-2.44	0.43	1.21	16.76
S-2	-1.96	0.77	**2.04**	-1.84	0.95	6.12	14.75
S-3	-1.27	-0.93	**1.96**	-2.20	1.93	2.42	17.90
S-4	-1.46	-0.74	**1.67**	-1.20	0.88	1.39	7.13

A similar result was reported in urban rivers such as the Langat River in Malaysia [5] and the river Hindon, India [8]. Enrichment factors revealed severe enrichment of Cd and Pb at downstream sites in river Ganga [39].

Moderately severe enrichment of Pb at S-4 and severe enrichment at all other sites were observed in terms of EF. Similarly, moderately severe enrichment of Zn was observed at S-2 and minor enrichment at other sites (Table 2). In the case of Ni, relatively low enrichment was noticed at S-3 site but no enrichment at other sites. These metals mean EF values were in the order of Pb > Zn > Ni. Overall, none of the riverbank sites were entirely free of anthropogenic enrichment, mainly Pb. Based on the values obtained for the geo-accumulation index and enrichment factor, two sites, i.e., S-2 and S-3, were found to be substantially contaminated. Both local point sources and non-point origins could be to blame for the contamination. In particular, S-2 and S-3 sites are located in highly urbanized areas and receive wastewater and urban wastes from mixed sources. In an earlier study in the Bharalu river sediments, the enrichment factor showed substantial loading of trace metals, and *I*_geo_ level indicated moderate to strong pollution [17]. *I*_geo_ of Zn showed strong to extreme contamination, and EF showed no enrichment to very severe enrichment in a polluted urban river in Bangladesh [41].

**Fig 3:**
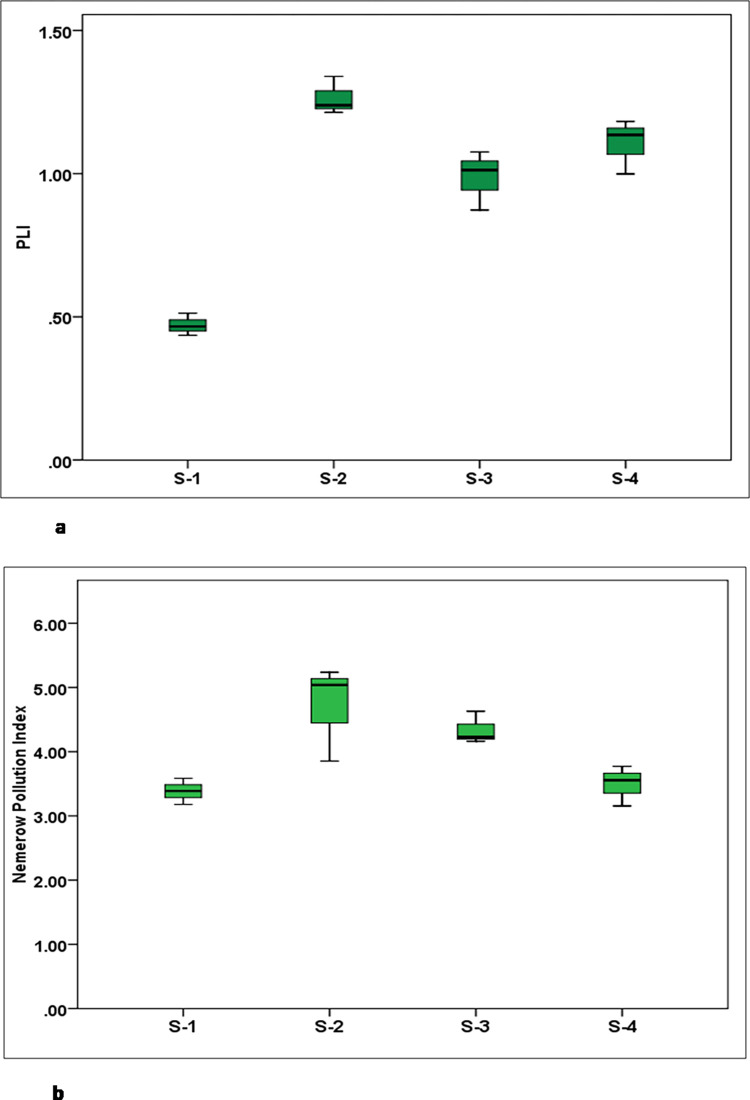
Box plot showing (a) Pollution Load Index (P.L.I) and (b) Nemerow’s Pollution index (P.I.N).

In our previous studies for the river Hindon, Ghaziabad, India, sources, such as urban and industrial discharges, were attributed to metal contamination in sediments [8,9,43]. Similarly, metal contamination in the river Gomti was also attributed to increased rapid urbanization and industrialization [44,45]. The values of PLI observed in the sediment of the Bharalu river are presented in Fig 3A. A PLI value of 1 means that the sediment is uncontaminated, while a value larger than 1 shows that the sediment is contaminated [29]. As shown in Fig 3A, PLI exceeded 1 at S-2 and S-4, indicating sediment contamination due to heavy metals. At S-2, the PLI value is close to 1 (0.98), whereas S-1 is relatively free from contamination. The overall order of sites based on PLI is S-2 > S-4 > S-3 > S-1. Site S-2 and S-3 mainly pass through densely populated residential and commercial areas, and S-4 is the downstream site. Urban discharges containing mixed liquid and solid waste from residential and commercial areas could be attributed to higher pollution at those sites. Site S-1 represents the river’s upper reach and is relatively away from the highly urbanized sites. Pb showed very high contamination at S-2 and considerable contamination at all other sites in terms of contamination factors. Zn showed moderate contamination at S-2 and minimal pollution at other sites. Ni and Fe showed low pollution in all sites. The EF, *I*_geo,_ and PLI values in the Tapti and Narmada rivers showed moderate pollution due to heavy metals [46]. Further, the study showed that enhanced metal concentrations in these river sediments were attributable to the urban and industrial direct discharge into the river [46]. In terms of *I*_geo_ and EF, the pollution level in the sediments of the Weihe River, China, showed non-pollution or slight pollution levels, while PLI showed moderate pollution levels at several sites [3].

The Nemerow’s Pollution Index (PIN) for river sediments is presented in Fig 3(B). PIN varied from 3.15 to 5.21, indicating ‘heavy’ pollution at all sites. Overall, the mean PIN (3.98) showed moderate contamination at the sampling locations. PIN values at different sites were in the order of S-2 >S-3 > S-4 >S-1. Metals were ranked according to their pollution indices as Pb > Zn > Ni > Fe. According to PIN values across all sites in the Shatt al-Arab River basin, the sediments are severely contaminated [47]. The sediments of the river Bharalu are contaminated with heavy metals, in particular with Pb is a matter of concern. Nevertheless, the Bharalu river’s stagnating level of water in recent decades is a severe problem. Stormwater and wastewater runoff from the public sector major refinery drains, and domestic wastewater from the entire basin contribute to pollution [48]. Previous studies reported severe contamination at Bharalumukh due to the dumping of city waste, where Bharalu joins the Brahmaputra river [18].

### 3.3. Potential sources of heavy metals

Correlation analysis and PCA are applied to determine the likely sources and relationships of heavy metals in sediments [8]. The results of the correlation analysis are depicted in Table 3.

**Table 3:** Metal-to-metal correlation coefficient matrix for metals in sediment samples of river Bharalu.

	Zn	Pb	Ni	Fe
Zn	1			
Pb	0.585*	1		
Ni	0.358	0.196	1	
Fe	0.372	-0.200	0.465	1
pH	0.116	0.005	0.288	0.593*

* Correlation is significant at the 0.05 level (2-tailed).

Except for Pb and Zn (r = 0.585), other metals found in the sediment matrix, such as Fe and Ni, showed no significant association with each other, implying different origins or sedimentological features. Based on their relationship, it is likely that Pb and Zn are from the same source and follow a similar pattern.

PCA with Varimax normalized rotation was done on the whole dataset to gain more trustworthy evidence regarding the correlations between the metals. Two major components (eigenvalues > 1) were recovered, representing 80.83 percent of the total variance (Table 4).

**Table 4:** Rotated component matrix of heavy metals.

Metals	PC1	PC2
Zn	0.452	**0.775**
Pb	-0.127	**0.954**
Ni	**0.752**	0.274
Fe	**0.920**	-0.121
Eigenvalues	1.941	1.292
% of Variance	48.52	32.30
% of Cumulative Variance	48.52	80.827

Bold indicates high loading values

Heavy metal PCA loadings and score graphs are provided in S1 Fig. Ni and Fe dominate the first component (PC1), which accounts for 48.52 percent of the overall variation (Table 4). Fe is naturally present in abundance. In the continental crust, Fe is the fourth most abundant element and the most abundant transition metal [49]. So, metals in PC1 may have dominant geogenic sources. PC2 is loaded particularly by Zn, and Pb explaining 32.31% of the total variance. The findings of this study revealed that, based on the correlation analysis, PC2 was consistent. Pb, Cu, and Zn deposition in sediments may be attributed to lead acid batteries, including other sources [50,51]. Heavy metals, including Zn and Pb enrichment in the Lijiang River, China, are linked to industrial emissions, metropolitan pollution, and natural sources [52]. The localities near river Bharalu are known for dumping residential waste and garbage into the river. It transports a significant amount of the city’s municipal and other garbage, as well as serves as an environmental drainage system for stormwater runoff [15]. The absence of an integrated system for sewage treatment and disposal in the city further threatens the river ecosystem [15,19]. It is a matter of concern that the contamination of the river Bharalu may also endanger the quality of the river Brahmaputra, often the principal source of consumptive water in the city.

### 3.4 Assessment of potential ecological risk

The average E^i^_r_ values (Table 5A) of the individual heavy metal in the Bharalu river subsided in the subsequent order: Pb> Ni> Zn. The maximum highest value of E_r_ (Pb) for S-4 was 100.4, indicating considerable risk, whereas sites-2 and 3 depicted low risk. The E^i^_r_ values of Ni and Zn showed very low risk at all the sites (Table 5A and 5B). Pb concentrations in Bharalu river sediments were generally higher than those reported in other rivers in India (S3 Table). However, the values of other metals (Ni, Zn, and Fe) are also high but manageable compared to Pb (S3 Table). It’s also worth noting that Pb is hazardous and non-biodegradable even at low doses [53]. Therefore, Pb^2+^, like other key divalent metals (Mn^2+^ and Zn^2+^), altered the conformance of nucleic acids, proteins, bacterial chemical movement restraints, and bacterial osmotic balance, all of which might negatively affect the ecosystem [53]. RI values indicate a low risk for all the studied sites (Tables 5B and S2). However, metals’ interactions with biological macromolecules, mainly Pb, can disrupt their normal metabolic and physiological processes, resulting in the poisoning and death of species [37].

**Table 5:** (a): Values of individual ecological risk (Ei_r_) for heavy metal in Bharalu river. **(b):** Values of ecological risk index (RI) for heavy metal in Bharalu river.

	**Ni**				**Zn**			
**Sampling site**	**Minimum**	**Maximum**	**Mean**	**St Dev**	**Minimum**	**Maximum**	**Mean**	**St Dev**
**S-1**	0.49	0.75	0.59	±0.11	0.27	0.38	0.34	±0.05
**S-2**	2.13	2.29	1.97	±0.35	2.48	2.63	2.56	±0.06
**S-3**	2.48	4.01	3.18	±0.63	0.67	0.87	0.79	±0.09
**S-4**	1.64	3.76	2.89	±0.91	0.23	1.01	0.90	±0.09
	**Pb**				**Fe**			
**S-1**	21.56	24.40	22.98	±1.16	ND	ND	ND	ND
**S-2**	25.10	34.78	31.07	±4.26	ND	ND	ND	ND
**S-3**	27.85	31.16	29.18	±1.43	ND	ND	ND	ND
**S-4**	20.83	25.1	23.21	±1.78	ND	ND	ND	ND

**Sampling site**	**Minimum**	**Maximum**	**Mean**	**St Dev**
**S-1**	22.32	25.53	23.91	±1.32
**S-2**	29.72	39.70	35.59	±4.67
**S-3**	30.99	36.04	33.15	±2.16
**S-4**	22.70	105.16	27.01	±2.77

St Dev: Standard Deviation, ND:Not Determined.

St Dev: Standard Deviation.

Cu showed the most serious potential ecological risk, while Cr and Pb showed low potential ecological risk in the sediment of the urban river [37]. Aproximately 70% of Cd samples lay above moderate risk, indicating that Cd is the primary factor causing an ecological hazard in the urban river-lake sediments [54]. Zn’s highest potential ecological risks occurred in sediments from a polluted urban river in central Bangladesh [41]. The high ecological risk was also observed in Shenyang City, China, in urban river sediments, indicating the importance of effective sewage management to protect the urban rivers’ environmental quality [33]. Due to the unavailability of toxicity coefficient values, we cannot calculate the ecological risk index (E_r_ and RI) for Fe.

## 4. Conclusion

This study investigated heavy metal contamination in the surface sediments of the human-impacted urban rivers using different approaches. Pollution indices in terms of PLI and PIN, showed moderate to heavy pollution, and Pb is of serious concern amongst all studied metals. Higher heavy metal pollution in the downstream sites could be attributed to urban discharges from residential and commercial areas. The correlation analysis and PCA suggested that Pb and Zn originated from anthropogenic sources, while Fe and Ni may have dominant geogenic sources. The ecosystem’s potential ecological risk ranged below low-risk values (RI<150), and Pb was found to be the major contributor to ecological risk. This is a preliminary investigation considering four metals. A more detailed and systematic study is needed along the entire stretch of this urban river. Detailed and regular monitoring of heavy metals and other pollutants in the urban river sediments is essential to take suitable measures for the protection of such river ecosystems as well as to protect the major rivers with which they confluence.

## Supporting information

S1 Fig PCA loadings and score plots of heavy metals in sediments. (TIF)

S1 Table Grade standards for *I*_*geo*_ and EF. (DOC)

S2 Table Various categories of potential ecological risk. (DOC)

S3 Table Heavy metals concentrations (mg/kg) in sediment of this study area and that reported in other rivers in India. (DOC)
