# Supplementary figures and images for: Heavy metals contamination in sediments of Bharalu river, Guwahati, Assam, India: A tributary of river Brahmaputra
Source: PLoS One. 2023 Apr 5;18(4):e0283665. doi: 10.1371/journal.pone.0283665 (PMC10075429; doi:10.1371/journal.pone.0283665)

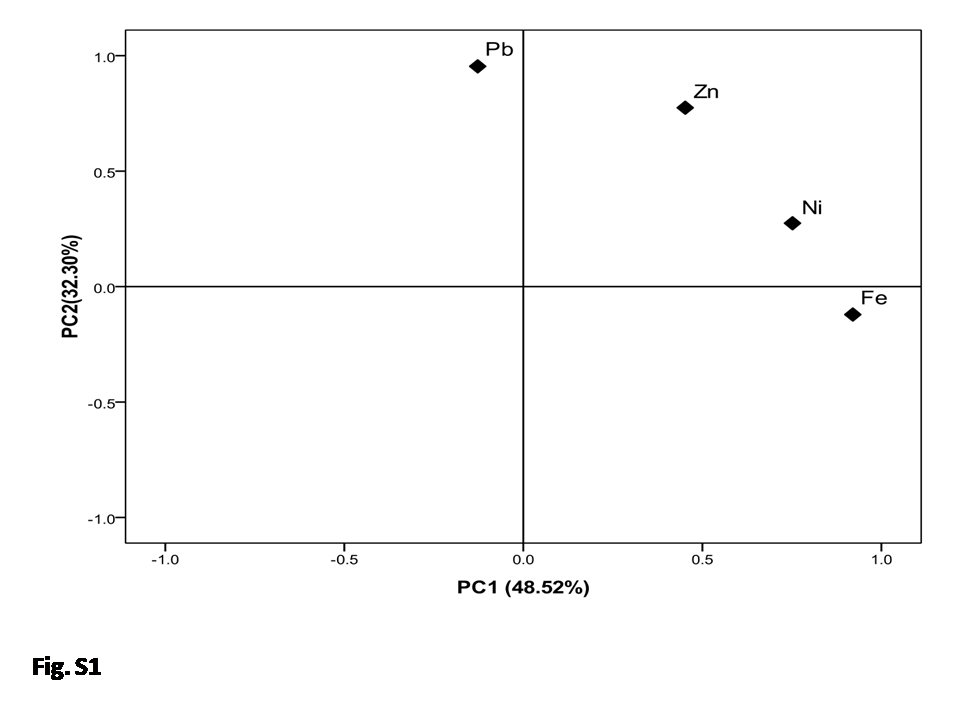

Supplement: S1 Fig — (TIF) [file pone.0283665.s001.tif]
