# Supplementary material for: Heavy metals contamination in sediments of Bharalu river, Guwahati, Assam, India: A tributary of river Brahmaputra
Source: PLoS One. 2023 Apr 5;18(4):e0283665. doi: 10.1371/journal.pone.0283665 (PMC10075429; doi:10.1371/journal.pone.0283665)
Supplement: S1 Table — (DOC) [file pone.0283665.s002.doc]

**Table S1.** Grade standards for *I_geo_* and EF

| *I_geo_* value | Class | Sediment quality | Grade standards for *I_geo_* (Förstner *et al*., 1993) |
| --- | --- | --- | --- |
| <0 | 0 | Practically Uncontaminated |  |
| >0-1 | 1 | Uncontaminated to moderate |  |
| >1-2 | 2 | Moderate |  |
| >2-3 | 3 | Moderate to strong |  |
| >3-4 | 4 | Strong |  |
| >4-5 | 5 | Strong to very strong |  |
| >5 | 6 | Very strong |  |
| <1 |  | No enrichment | (Birth; 2003) |
| 1-3 |  | Minor enrichment |  |
| 3-5 |  | Moderate enrichment |  |
| 5-10 |  | Moderately severe enrichment |  |
| 10-25 |  | Severe enrichment |  |
| 25-50 |  | Very severe enrichment |  |
| >50 |  | Extremely severe enrichment |  |

Forstner U, Ahlf W, Calmano W. Sediment quality objectives and criteria development in Germany. Water Sci Technol. 1993; 28: 307–316. <https://doi.org/10.2166/wst.1993.0629>.

Birth G, 2003. A scheme for assessing human impacts on coastal aquatic environments using sediments. In: Woodroffe, C.D., Furness, R.A., (Eds.), Coastal GIS.
