# Supplementary material for: Heavy metals contamination in sediments of Bharalu river, Guwahati, Assam, India: A tributary of river Brahmaputra
Source: PLoS One. 2023 Apr 5;18(4):e0283665. doi: 10.1371/journal.pone.0283665 (PMC10075429; doi:10.1371/journal.pone.0283665)
Supplement: S2 Table — (DOC) [file pone.0283665.s003.doc]

**Table S2:** Various categories of potential ecological risk (Liu, et al., 2021)

| **E_r_** | **Individual ecological risk** | **RI** | **Potential ecological risk to the environment** |
| --- | --- | --- | --- |
| E_r_<40 | Low risk | RI<150 | Low risk |
| 40≤E_r_<80 | Moderate risk | 150≤RI<300 | Moderate risk |
| 80≤E_r_<160 | Considerable risk | 300≤RI<600 | Considerable risk |
| 160≤E_r_<320 | Higher risk | RI>600 | High risk |
| E_r_≥320 | Extremely high risk |  |  |

Liu D, Wang J, Yu H, Gao H, Xu W. Evaluating ecological risks and tracking potential factors influencing heavy metals in sediments in an urban river. Environmental Sciences Europe, 2021; 33(1): 1–13. <https://doi.org/10.1186/s12302-021-00487-x>.
