# Supplementary material for: Heavy metals contamination in sediments of Bharalu river, Guwahati, Assam, India: A tributary of river Brahmaputra
Source: PLoS One. 2023 Apr 5;18(4):e0283665. doi: 10.1371/journal.pone.0283665 (PMC10075429; doi:10.1371/journal.pone.0283665)
Supplement: S3 Table — (DOC) [file pone.0283665.s004.doc]

**Table S3.** Heavy metals concentrations (mg/kg) in sediment of this study area and that reported in other rivers in India.

| **Rivers** | **Pb** | **Fe** | **Zn** | **Ni** | **References** |
| --- | --- | --- | --- | --- | --- |
| Bharalu River, Assam, India | 106.4 | 19548.2 | 108.9 | 29.4 | Present Study |
| Gomti river | 28.47 | - | 90.03 | 18.97 | (Gupta et al., 2015) |
| Ganga | 26.7 | 31,988.6 | 67.8 | 26.7 | (Pandey and Singh, 2017) |
| Cauvery | 4.3 | 11,144 | 93.1 | 27.7 | (Raju et al., 2012) |
| Narmada | 13.9 | 89,577 | 196.2 | 200.3 | (Sharma and Subramanian,2010) |
| Tapti | 25.0 | 91,128 | 216.7 | 205.5 | (Sharma and Subramanian,2010) |
| Ghaghara River, India | 12.5 |  | 15.4 | 20.4 | (Singh et al., 2017) |
| Indian average | 11.2 | 29,983 | 16 | 37 | (Subramanian et al., 1985) |
| World average | 150 | 48,000 | 350 | 90 | (Martin and Meybeck, 1979) |

**Reference**

Gupta SK, Chabukdhara M, Singh J, Bux F. Evaluation and potential health hazard of selected metals in water, sediments, and fish from the Gomti River. Human Ecol Risk Assess. 2015; 21: 227–240. <https://doi.org/10.1080/10807039.2014.902694>.

Pandey J., Singh, R. Heavy metals in sediments of Ganga River: up- and downstream urban influences. Appl Water Sci. 2017; 7: 1669–1678. https://doi.org/10.1007/s13201-015-0334-7.

Raju, K.V., Somashekar, R., Prakash, K () Heavy metal status of sediment in river Cauvery, Karnataka. Environ Monit Assess. 2012; 184(1): 361–373. <https://doi.org/10.1007/s10661-011-1973-2>

Sharma SK, Subramanian V. Source and distribution of trace metals and nutrients in Narmada and Tapti river basins, India. Environ Earth Sci. 2010; 61: 1337–1352. doi:10.1007/s12665-010-0452-3.

Singh H, Pandey R, Singh HK, Shukla D. Assessment of heavy metal contamination in the sediment of River Ghaghara, a major tributary of the River Ganga in Northern India. Appl Water Sci. 2017; 7(7): 4133–4149. [10.1007/s13201-017-0572-y](http://dx.doi.org/10.1007/s13201-017-0572-y)

Subramanian V, Van’t Dack L, Van Grieken R. Chemical composition of river sediments from the Indian Sub-continent. Chemical Geol. 1985; 48: 271–279. <https://doi.org/10.1016/0009-2541(85)90052-X>

Martin. J.M., Meybeck, M. Elemental mass balance of material carried by major world rivers. Marine Chem. 1979; 7: 173–206. <https://doi.org/10.1016/0304-4203(79)90039-2>
